# Supplementary material for: Proxy-based model to assess the relative contribution of ballast water and biofouling’s potential propagule pressure and prioritize vessel inspections
Source: PLoS One. 2021 Jul 1;16(7):e0247538. doi: 10.1371/journal.pone.0247538 (PMC8248655; doi:10.1371/journal.pone.0247538)
Supplement: S1 Flowchart — (PDF) [file pone.0247538.s003.pdf]

# STEP-BY-STEP PROCESS

## POPULATION PRE-ASSESSMENT – One time

1. Define your population using previous data (any time frame). Input your data in the "*Population data*" tab in the template provided in **S4**.
2. Calculate **TWSA** using the regression equations presented on Table 1 and **Equation 1**. Use the script on **S3** for the calculations.
3. Identify the **medTWSAind** and **medBWDind.**

## ARRIVALS PRIORIZATION – Daily

1. Calculate **PPP score** to prioritize daily arrivals: Input the "*Arrivals data*" in the template provided in **S4.**
2. Calculate **TWSA** as described for the **Population pre-assessment**, if not available already.
3. Use **Equation 2** (included in the script on **S3**) to calculate the **PPP score**.

## POPULATION PATTERNS – Retroactively

Calculate **Cumulative PPP score** on specific locations and/or vessel type by summing individual **PPP scores**.
