# Supplementary material for: Proxy-based model to assess the relative contribution of ballast water and biofouling’s potential propagule pressure and prioritize vessel inspections
Source: PLoS One. 2021 Jul 1;16(7):e0247538. doi: 10.1371/journal.pone.0247538 (PMC8248655; doi:10.1371/journal.pone.0247538)
Supplement: S1 Script — (DOCX) [file pone.0247538.s004.docx]

S3_PPP_Calculations.R

Lina.Ceballos

2021-05-05

library(readxl)

#import Population data
Population_data<-read_excel("S4_Data_template_PPP_Score_Calculations.xlsx", sheet="Population_data")

#____Calculate TWSA using the regression equations for each vessel type

#separate by vessel type
subset_General<-subset(Population_data, Population_data$VesselType=="General")
subset_Passenger<-subset(Population_data, Population_data$VesselType=="Passenger")
subset_RO_RO<-subset(Population_data, Population_data$VesselType=="RO-RO")
subset_Bulker<-subset(Population_data, Population_data$VesselType=="Bulker")
subset_Container<-subset(Population_data, Population_data$VesselType=="Container")
subset_Tanker<-subset(Population_data, Population_data$VesselType=="Tanker")

#calculate TWSA by calculating WSA and adding the niche area proportion
subset_General$TWSA<-(('^'(subset_General$GrossTon, 0.5728))*20.02)+(('^'(subset_General$GrossTon, 0.5728))*20.02)*0.09
subset_Passenger$TWSA<-((('^'(subset_Passenger$GrossTon, 0.6951))*5.46)*0.27)+(('^'(subset_Passenger$GrossTon, 0.6951))*5.46)
subset_RO_RO$TWSA<-(('^'(subset_RO_RO$GrossTon, 0.5309))*25.04)+(('^'(subset_RO_RO$GrossTon, 0.5309))*25.04)*0.09
subset_Bulker$TWSA<-(('^'(subset_Bulker$GrossTon, 0.6294))*15)+(('^'(subset_Bulker$GrossTon, 0.6294))*15)*0.07
subset_Container$TWSA<-(('^'(subset_Container$GrossTon, 0.6501))*10.66)+(('^'(subset_Container$GrossTon, 0.6501))*10.66)*0.09
subset_Tanker$TWSA<-(('^'(subset_Tanker$GrossTon, 0.6105))*17.57)+(('^'(subset_Tanker$GrossTon, 0.6105))*17.57)*0.08

Population_data<-rbind(subset_General,subset_Passenger, subset_RO_RO, subset_Bulker, subset_Container, subset_Tanker)

#____Calculate the PPP


#find Median BWD of the discharging population
just_disch<-subset(Population_data, BWDind>0)
medBWD<-median(just_disch$BWDind)
medBWD

## [1] 2282.1

#find Median TWSA of the population
medTWSA<-median(Population_data$TWSA)
medTWSA

## [1] 12303.17

#____Calculate relative PPP for BW and BF in the arrivals data.
# Import arrivals data
arrivals<-read_excel("S4_Data_template_PPP_Score_Calculations.xlsx", sheet="Arrivals_data")
#divide each component by the population median
arrivals$rBW<-arrivals$BWDind/medBWD
arrivals$rBF<-arrivals$TWSA/medTWSA

#calculate PPP Score by adding the relative BW score and relative BF score
arrivals$PPP_score<-arrivals$rBW+arrivals$rBF
